# Intervention and efficacy of advance care planning for patients in intensive care units and their families: a scoping review protocol

**DOI:** 10.1002/nop2.722

**Published:** 2020-11-30

**Authors:** Kanako Yamamoto, Junko Hayama, Kazuhiro Nakayama, Yuki Yonekura, Erika Ota

**Affiliations:** ^1^ Graduate School of Nursing Science Nursing Infomatix St. Luke's International University Tokyo Japan; ^2^ Graduate School of Nursing Science Global Health Nursing St. Luke's International University Tokyo Japan

**Keywords:** advance care planning, critical care, intensive care, nurses, nursing, systematic review

## Abstract

**Aim:**

This scoping review aims to elucidate the effectiveness of advance care planning interventions for patients entering the intensive care unit and their families.

**Design:**

Scoping review of relevant literature from January 2000–March 2020.

**Methods:**

This review includes studies undertaken in intensive care units that focus on patients older than 18 years or their families. The review will be conducted in accordance with the PRISMA‐P guideline. The PubMed, EMBASE, CINAHL, BNI, PsycINFO and ICHUSHI databases and the Cochrane Library will be searched for both published and unpublished articles. Two independent reviewers will examine the list of remaining titles and summarize and identify articles that meet the inclusion criteria.

**Results:**

It has long been taboo to consider end‐of‐life care when in intensive care unit. However, promoting advance care planning, even in patients who are in the intensive care unit, is important and it may help support the patient's need for autonomy.

## 1 INTRODUCTION

Recent advances in medical care have increased the health and medical care options of many older adults and the chances of recovery of patients with multiple diseases who undergo invasive treatments (Flaatten et al., 2017). Many of these patients are treated in intensive care units (ICUs). Treatment in the ICU is usually predicated on active treatment; however, depending on the situation, continued active treatment may not always benefit the patient. During the treatment, it may become necessary to switch to end‐of‐life care. End‐of‐life care is medical treatment aimed at improving and optimizing the quality of life of patients with serious, incurable illnesses and their families. The opportunities to support end‐of‐life care are expected to increase drastically in the future. Therefore, it is necessary for all individuals living in ageing societies to think about how to live at the end of their life, regardless of their current age. Furthermore, it is important for healthcare providers to respect patients' preferences for end‐of‐life care, as part of providing quality care (Allison & Sudore, 2013).

One solution to this problem is the advance care planning (ACP) approach. ACP is defined as ‘an approach to think in advance about the medical care that the patient wants at the end of their life and to repeatedly discuss and share the information with medical care providers’ (Ministry of Health, Labor, & Welfare, 2018). ACP is essentially a life plan for people to have autonomy in their own lives in all circumstances. However, current ACP interventions in hospitals focus on patients nearing the end of life, which may indicate that these are deviating from their original objectives.

### 1.1 Background

Until around 20–30 years ago, the concept of ‘life‐prolonging treatment’ was not clear and was only included in the broad understanding of ‘treatment’ (Nitta, 2016). At a time when a patient's dignity was emphasized, providing medical care to preserve life was seen as the best decision. However, trends of the times and circumstances are changing little by little and more and more people are considering that they would prefer to end their lives with dignity, rather than with life‐prolonging treatment. Because of these changes, ACP is being addressed worldwide as a decision support initiative in end‐of‐life care.

End‐of‐life care is for all generations and it is recommended that ACP, which is at the core of end‐of‐life care, be implemented across all age groups (Sumida, 2019). Multigenerational ACP can be classified into three categories according to the health status and life stage of the individual (Wilkinson & Shugarnab, 2017). The first stage involves ACP as a form of value education for healthy individuals. The second stage involves ACP in the context of community medicine for older adults and patients with chronic diseases. The third stage involves ACP in critical care and terminal care settings. Individuals in the third stage are patients with severe illnesses or a limited prognosis. The introduction of ACP is now underway around the world, and many current ACP studies are aimed at older adults in nursing homes, cancer patients and patients with chronic illnesses. In other words, ACP targets individuals in the second or third stage (Kuusisto et al., 2020; Spacey et al., 2019; Schichtel et al., 2020).

Appropriate timing for the introduction of ACP is considered to be when the patient's course of treatment changes, when the condition of the patient worsens, or when there is a significant decline in their physical function (Jensen et al., 2015). Further, a study in Switzerland reported that a sizeable proportion (42%) of physicians suggested that the best time to discuss ACP is prior to a highly invasive surgery (Gigon et al., 2015). Likewise, it may be appropriate to consider ACP for postoperative patients who enter the ICU following a highly invasive surgery. Considering the advances in minimally invasive surgical procedures in Japan in recent years, older patients with multiple chronic illnesses face increased opportunities to enter the ICU. However, although the surgery itself might not be highly invasive, such patients have low recovery capability and often become frail. In such cases, postoperative complications are most likely to occur and the patients may lose consciousness or die (Beggs et al., 2014). This is one of the most undesirable consequences of treatment for both patients and healthcare practitioners and is an important potential problem.

In many cases, patients treated in the ICU often lack the ability to make decisions due to impaired consciousness or the use of sedatives. Therefore, their family members must take charge of making proxy decisions for the patients. However, this can be a highly traumatic experience for the family members. One study found that a third of family members suffer from post‐traumatic stress disorder after the death of a patient in the ICU, which may partly be related to the need to make proxy decisions (Azoulay et al., 2005). Proxy decision‐makers are often overburdened. Major factors that inhibit proxy decision‐makers from making decisions are a lack of understanding of the patient's desired treatment and preferences and the patient's own judgements about care.

In addition, many ICU physicians and nurses find it difficult to explain the end‐of‐life situation to their patients (Hilton et al., 2013; Marufuji et al., 2017). Considering this background, even though the need for ACP support for ICU patients is recognized, there are challenges to overcome. For example, in many perioperative contexts, ‘recovery’ by means of treatment is often presupposed. Therefore, there tends to be less motivation to study the effectiveness of ACP.

A systematic review was conducted to investigate the possibility of ACP in perioperative patients who were considering aggressive therapy; however, this did not include perioperative patients (Aslakson et al., 2015). Moreover, many ACP studies have established whether in certain outcomes, ICU admission can be avoided (Ashana et al., 2019; Khandelwal et al., 2015). There have been very few studies of ACP among patients entering the ICU who lack decision‐making ability. Therefore, it is also important to determine the efficacy of ACP in patients who are in ICUs or who are perioperative.

An existing systematic review and meta‐analysis demonstrated the efficacy of ACP in all settings (Houben et al., 2014). This review included only one study of patients admitted to ICU (Song et al., 2005). In recent years, the need for ACP for patients admitted to ICU has been pointed out (Ramachenderan & Auret, 2019). In the future, it may also be necessary to update the evidence to clarify the necessity and merit of ACP for patients entering the ICU.

A scoping review is an exhaustive search of existing literature pertaining to the topic of interest. The selected articles are classified by their nature, characteristics and quantity. Thereafter, definitions and conceptual boundaries concerning the topic or field are clarified. ACP is still in its developmental stage; therefore, a scoping review would enable the researchers to answer a wide range of exploratory questions. There currently are no systematic scoping reviews or a scoping review protocol on this topic. Therefore, this study aims to assess the effectiveness of ACP for patients in the ICU and their families.

### 1.2 Research questions

This review aims to address the following research questions:

1. What are the effects of introducing ACP to patients in the ICU?
2. What are the effects of introducing ACP to families of patients in the ICU?
3. What are the benefits of and needs pertaining to ACP intervention for patients admitted to ICU during the perioperative period and their families?

## 2 THE STUDY

### 2.1 Design

This study uses the scoping review method. The purpose of a scoping review is to review a body of literature, identify gaps in existing knowledge and define concepts or explore research practices (Munn et al., 2018). The review process will follow the framework proposed by Arksey and O'Malley (2005) and advanced by Levac et al. (2010). The framework involves the following stages: (a) identifying the research question(s), (b) identifying relevant studies, (c) selecting the study to undertake, (d) charting the data and (e) summarizing and reporting the results. This review will be conducted in accordance with the PRISMA‐ScR (Tricco et al., 2018). It does not attempt to perform a critical scrutiny but simply aims to yield a narrative integration of the topic. ‘Scoping reviews serve to synthesize evidence and assess the scope of literature on a topic and help determine whether a systematic review of the literature is warranted’ (PRISMA, 2015).

### 2.2 Method

#### 2.2.1 Inclusion criteria

This scoping review will include studies that collected data from participants meeting the following criteria:

1. The years of publication of the literature reviewed range from January 2000–March 2020.
2. Regarding language, papers written in English and those written in Japanese that the authors can review accurately are to be included.
3. This scoping review will examine major quantitative and qualitative studies. Quantitative studies will include intervention studies such as randomized and non‐randomized controlled trials and pre–post intervention studies. Analytical observational studies will exclude prospective and retrospective cohort studies and case–control studies. Qualitative studies will include qualitative descriptive studies but are not limited by the study design.
4. Studies on adult patients (≥18 years) who entered the ICU were included. Patients younger than 18 years, patients who were being treated for mental health conditions, terminally ill patients, new mothers and patients in nursing homes or hospices were excluded.
5. Studies with participants who were family members or representatives of a patient who entered the ICU were included. For this review, a family member is defined as the patient's surrogate decision‐maker, regardless of their blood relationship.
6. Studies involving only healthcare professionals were excluded.

### 2.3 Concept

Advance care planning includes advance directives (ADs), the living will (LW) of the patient, orders to not attempt resuscitation (DNAR) and a proxy directive (Adach, 2019). ADs are documented through an ACP discussion and include proxy instructions as well as the LW of an individual. The LW of the patient also includes DNAR orders.

### 2.4 Context

This review targets hospital ICUs for adults. The following settings will be excluded from the review: emergency room and acute care units that are not intensive care.

### 2.5 Search strategy

Search uses a two‐step process. The first step is a limited search of the PubMed and EMBASE databases (Appendix S1). The search terms used in this study are chosen with the help of academic librarians. The research team also uses an iterative process to identify search terms. The second step is to add five electronic databases to search: BNI, CINAHL, ICHUSHI (for research in Japan), PsycINFO and The Cochrane Library. In addition, searches for unpublished studies containing grey literature are conducted in the OpenGrey and Trip databases. If necessary, we will modify our search strategy to improve the amount and relevance of results.

### 2.6 Analysis

The database search results will be exported to Rayyan (https://rayyan.qcri.org/welcome). Duplicate documents will be removed from the total list of citations obtained. Two independent reviewers will examine the list of remaining titles and abstracts and identify studies that meet the selection criteria. Full‐text articles that meet the inclusion criteria will be read and reviewed by two reviewers. These steps will be performed to ensure the accuracy of the selected studies. Disagreements between reviewers in the abstract or full‐text screening phase are resolved through discussion until an agreement is reached. A third reviewer is used to resolve differences, if necessary. Finally, all reviewers check the selected documents. We will also create a PRISMA research flow diagram to illustrate the search results. As recommended by Levac et al. (2010), we will provide narrative synthesis to explain the findings.

### 2.7 Data extraction

The data extraction form contains the following items: data on author, publication date, country of origin, purpose, population, type (ACP or AD), study design, setting (ICU), intervention, results and main outcomes will be included (Tables 1 and 2).

**TABLE 1:** Draft charting Table 1

Study #	First author	Publication date	Country of origin	Purpose	Type	Population	Study design
1							
2							
3							

**TABLE 2:** Description of ACP intervention

Study #	Setting (ICU)	Intervention	Results and Main outcome
1			
2			
3			

### 2.8 Data presentation

The relevant data from extracted articles will be summarized in tabular form using an Excel sheet for the review.

### 2.9 Ethics

This study is a literature study and does not require ethical approval. Reviewers explicitly declare conflicts of interest with all studies that are included or not included in the review.

### 2.10 Limitations

Since the setting of this study is an intensive care unit, which is the area where the study was initiated recently, it is expected that the number of documents will be less.

Furthermore, it is difficult to identify higher evidence due to the small number of documents and the difficulty of systematic review.

## 3 DISCUSSION

Intensive care units are recognized as places where lifesaving medical care is provided. Therefore, considering end‐of‐life care for patients in ICU has long been taboo. For example, healthcare providers are aware that patients who choose aggressive treatment, such as surgery, should not confirm their intention to receive treatment when they enter end‐of‐life care. Similarly, patients may worry that they may not have access to appropriate medical care when they are thinking about their end‐of‐life wishes and communicating them to their healthcare provider. We believe this is why ACP efforts around the world have not progressed as expected. It is important that patients feel that the treatment they are receiving is their own choice and that they have autonomy over their life. The treatment patients choose to receive depends on them alone. Patients will choose to receive medical care that ensures that they receive appropriate treatment and that their right to autonomy is adhered to. We believe that identifying ACP support and its effectiveness for patients who are scheduled to be admitted to ICU and those who are already admitted to ICU may provide clues regarding how to protect patient autonomy and to consider effective support methods.

## 4 CONCLUSION

In this scoping review, we hope that ACP will not be end‐of‐life specific but will be used as a tool for continuous use. In addition, it may shed light on the advantages and disadvantages of introducing ACP in the ICU and expect it to aid in clinical intervention.

## CONFLICT OF INTEREST

The authors declare no conflict of interest.

## AUTHOR CONTRIBUTIONS

All five authors have made substantial contributions to conception and design, or acquisition of data, or analysis and interpretation of data. KY was primarily involved in drafting the manuscript and with significant input from YY and JH and KN and EO in revising it critically for important intellectual content. All five authors have agreed to be accountable for all aspects of the work in ensuring that questions related to the accuracy or integrity of any part of the work are appropriately investigated and resolved.

## Supporting information

Appendix S1

## Data Availability

No data sets were generated or analysed during the current study.
