# Supplementary material for: Intervention and efficacy of advance care planning for patients in intensive care units and their families: a scoping review protocol
Source: Nurs Open. 2020 Nov 30;8(2):997–1001. doi: 10.1002/nop2.722 (PMC7877163; doi:10.1002/nop2.722)
Supplement: Supplementary file 1 — Appendix S1 [file NOP2-8-997-s001.docx]

**Appendix I: Search strategy for EMBASE and PubMed**

Search strategy for EMBASE and PubMed.

**A. EMBASE**

PICO Search

Population: Intensive care unit/ OR / Perioperative. ti.ab.

Intervention: advance care planning/ OR / living will. ti.ab.

**B. PuBMed**

1. Intensive Care Unit/ AND/ Advance Care Planning/ AND/ (Perioperative Period/ OR/ Perioperative). ti.ab.

2. Intensive Care Unit/ AND/ ((Advance Directives) OR (Advance Directives)). ti.ab.

3. Intensive Care Unit/ AND Living Will. ti.ab.

4. Intensive Care Unit / AND ((Advance Directives) OR (Advance Directives))/ AND / (Perioperative period OR Perioperative). ti.ab.

5. Intensive Care Unit/ OR/ Living Will AND (Perioperative Period) OR (Perioperative) ti.ab.

6. Intensive Care Unit/ AND / Advance Care Planning AND (Perioperative Period) OR (Perioperative). ti.ab.
